
JOURNAL INFORMATION
==============================
NLM Title Abbreviation: Buffalo Med J Mon Rev Med Surg Sci
NLM Journal ID: 0004276

The Buffalo Medical Journal and Monthly Review of Medical and Surgical Science

Publisher: Biomedical Journal Digitization Project

ARTICLE INFORMATION
==============================
PMCID: PMC8936133
PMID: 35376956
Article version: 1
Subjects: Editorial Department

Letter to Prof. Hamilton

Leonard A. M.
Print publication date: 1858 Jun
Volume: 14
Issue: 1
First page: 62
Last page: 62
License: This work is free of known copyright restrictions. For more information, please see PMC Back Issue Digitization.
License URL: https://creativecommons.org/publicdomain/mark/1.0/

==============================

LOCKPORT, May 31st, 1856.

PROF. HAMILTON:

Dear Sir,—I send you the followfng report of a case that occurred in my practice, and which may possess interest enough to entitle it to a more extended publicity. You are at liberty to do with it as you see fit:

In the summer of 1853, Mr. Monroe Levally, wagon-maker, aged 46, called at my office and requested me to examine his arm. I did so, and found immediately over the point of the olecoron process an encysted tumor of the size of a robin's egg, and which made its appearance a few days previous. I made a few incisions into the sack with an abcess lancet, and obtained about ʒiii of a thick fluid. Introduced into the sack a pledgit of lint, and in a few days a perfect cure was effected.

But the next week I was not a little surprised, when he returned, with *another tumor* on the *other* arm, an exact counterpart of the prevlous one in every particular. It made its appearance in the same manner; in locality it was the same, (except on the other arm,) of the same size and form. I treated in the same manner, and the result was the same.

How far this goes to prove the existence of a sympathitic action between the corresponding points of the human system, we leave for others to decide; but it does certainly go far to prove that, under certain circumstances, one elbow at least has some sympathy with the other.

Yours, ever,

A. M. LEONARD.

DR. FLINT,—"Symmetrical diseases" are known to occur often in eruptive affections, and occasionally in rheumatic and syphylitic affections, but this is the only example which I have known where an encysted tumor, or a true tumor of any kind, has illustrated this curious law of affinities between opposite portions of the body.

Yours,

F. H. HAMILTON.

---

*The Savannah Journal of Medicine.*—We have received the first number of this Journal, issued in May. The editors are J. S. Sullivan, M. D., Josiah Harris, M. D., Prof. of Physiology in the Savannah Medical College, and R. D. Arnold, M. D., Prof. of Theory and Practice of Medicine in the Savannah Medical College, Associate Editor. This Journal will be issued bi-monthly, and contains seventy-two pages. The first number pre-
